# Cs_2_SnCl_6_: To Emit or to Catalyze? Te^4+^ Ion Calls the Shots

**DOI:** 10.1002/advs.202302706

**Published:** 2023-08-09

**Authors:** Haiwen Wei, Jikai Sun, Xin Mao, Honglei Wang, Zhen Chen, Tianxin Bai, Pengfei Cheng, Ruiling Zhang, Bing Jin, Panwang Zhou, Feng Liu, Keli Han

**Affiliations:** ^1^ Institute of Molecular Sciences and Engineering Institute of Frontier and Interdisciplinary Science Shandong University Qingdao 266237 P. R. China; ^2^ School of Chemical Engineering Dalian University of Technology Dalian 116024 P. R. China; ^3^ State Key Laboratory of Molecular Reaction Dynamics Dalian Institute of Chemical Physics Chinese Academy of Science Dalian 116023 P. R. China

**Keywords:** CO_2_ photocatalytic reduction, lead‐free tin perovskites, perovskite alloying, perovskite photocatalysis, Te^4+^‐doped Cs_2_SnCl_6_

## Abstract

A low concentration of Te^4+^ doping is found to be capable of endowing the lead‐free Cs_2_SnCl_6_ perovskites with excellent photoluminescence quantum yield (PLQY), while further increasing Te^4+^ concentration leads to PLQY deterioration. The mechanism behind the improved PLQY is intensively studied and reported elsewhere. However, little work is conducted to understand the decreased PLQY at high doping levels and to explore its implications for non‐PL‐related applications. Here, it is demonstrated that the Te^4+^‐incorporated Cs_2_SnCl_6_ can be promising candidate for efficient CO_2_ photocatalysis. An optimum photocatalytic performance is achieved when Te^4+^ concentration reaches as high as 50%, at which point significant PL quenching has occurred. Through a detailed spectral characterization, such concentration‐dependent functionality is attributed to systematic changes in both electronic and local crystal structure, which allow a robust regulation of excitation energy relaxation channels. These findings expand the scope of available photocatalysts for CO_2_ reduction and also inform synthetic planning for the preparation of multifunctional Pb‐free metal halide perovskites.

## Graphical abstract

A functional switch of Cs_2_SnCl_6_ perovskite between light emission and CO_2_ photocatalysis is enabled by controlling the amount of Te^4+^ in the lattice structure, which plays a key role in regulating both electronic and local crystal structure of the host, and thus allows a robust control over excitation energy relaxation pathways.

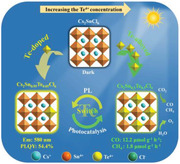

## 1 Introduction

As an emerging and promising candidate for solar energy conversion, all‐inorganic lead (Pb)‐free metal halide perovskite derivatives, such as, Cs_2_AgInCl_6_, Cs_3_Sb_2_Br_9_, and (CH_3_NH_3_)_3_Bi_2_I_9_, etc., have attracted great interest for various applications, such as photocatalysis, light‐emitting diodes, and solar cells.^[^ ^1^ ^]^ Compared to those Pb‐based halide perovskites (e.g., CsPbI_3_ and CH_3_NH_3_PbBr_3_), these environmentally friendly materials are even more stable under ambient conditions and also possess unique photophysical properties that are derived from their peculiar structures, such as the intrinsic quantum confinement and self‐trapped excitons (STEs).^[^ ^2^ ^]^ However, most Pb‐free perovskite alternatives suffer from a serious nonradiative loss of photoexcited carriers because of their indirect transition band gaps,^[^ ^3^ ^]^ defect intolerance,^[^ ^4^ ^]^ and/or parity/spin‐forbidden transition,^[^ ^5^ ^]^ which significantly limits their potential for high‐performance solar energy conversion.

Recently, isovalent/heterovalent doping or alloying has become one of the most commonly employed approaches to regulate the intrinsic optoelectronic properties of Pb‐free metal halide perovskites. For instance, Tang et al. alloyed Na^+^ cations into Cs_2_AgInCl_6_ double perovskite to break the inversion symmetry‐induced parity‐forbidden transition.^[^ ^6^ ^]^ Alloyed Cs_2_Ag_0.6_Na_0.4_InCl_6_ presented an enhanced photoluminescence quantum yield (PLQY) by three orders of magnitude compared to the pristine one. Further Bi^3+^ doping improved crystal perfection and promoted exciton localization, leading to an optimized PLQY of 86%; Li and co‐workers reported that the Sb^3+^‐alloyed Cs_3_Bi_2_Br_9_ perovskites exhibited improved catalytic performance for activating benzylic C─H bond as a result of the enhanced charge separation and energy transfer.^[^ ^7^ ^]^ In a recent report from our group, Sb^3+^ alloying strategy was also employed to regulate excitation energy transfer pathways in Cs_4_MnBi_2_Cl_12_ perovskites for efficient CO_2_ photoreduction and water oxidation.^[^ ^8^ ^]^ As an important kind of Pb‐free perovskite alternatives, vacancy‐ordered Cs_2_SnCl_6_ has recently received increasing attention for light‐emitting materials and photodetectors owing to their excellent air stability and tunable electronic properties.^[^ ^9^ ^]^ The pristine Cs_2_SnCl_6_ is substantially nonemissive because of its intrinsic Sn vacancy trapping defects and dipole‐forbidden transition.^[^ ^10^ ^]^ In recent years, great efforts have been devoted to improve their luminescent properties, such as doping with Te^4+^ and Sb^3+^, which allows a significant improvement in PLQY (95.6% for Te^4+^ and 41.6% for Sb^3+^, respectively).^[^ ^11^ ^]^ Such PL enhancement has been widely studied and mainly ascribed to the formation of additional luminescent centers (e.g., [TeCl_6_]^2−^ and [SbCl_6_]^3−^ octahedra) and the Jahn–Teller‐like STEs. It is generally observed that luminescence efficiency of the doped compounds depends highly on dopant concentration and usually an optimum PLQY is achieved with a very low doping level (≈1%);^[^ ^12^ ^]^ further increase in dopant concentration will lead to a severe PLQY deterioration. While a great deal of studies has been carried out to reveal mechanisms behind the improved PLQY, less attention has been paid to the scenarios where dopant concentration is overabundant, moreover, explorations for their non‐PL‐related applications remain largely uncharted.

In this work, we investigated how variation in Te^4+^ concentration influences photophysical properties of Cs_2_SnCl_6_ perovskites and how it governs their performance toward light emission and photocatalytic CO_2_ reduction. We found that a high level of Te^4+^ incorporation quenches PL of the material, but, meanwhile, it unlocks possibilities for efficient CO_2_ photocatalytic reduction. Such concentration‐triggered switching from PL to photocatalysis is revealed to be mainly associated with the change in both electronic and local crystal structure of the host, which allows a robust regulation of excitation energy relaxation pathways. The resulting optimally synthesized Cs_2_Sn_0.5_Te_0.5_Cl_6_ perovskites exhibit a high CO evolution rate of 28.6 and 3.3 µmol g^−1^ h^−1^ for CH_4_ with total electron consumption reaching 83.6 µmol g^−1^ h^−1^, superior to most other Pb and Pb‐free halide perovskites.

## 2 Results and Discussion

**Figure 1:**
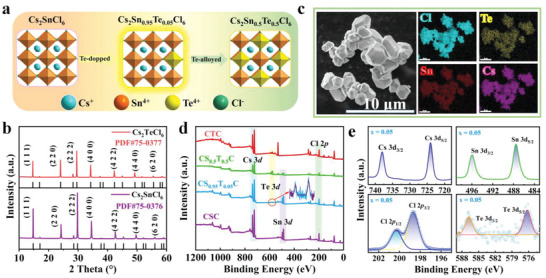
a) Crystal structure diagram of Cs_2_SnCl_6_ and Te^4+^‐doped/alloyed crystals. b) Powder XRD patterns of the obtained crystals and the standard XRD patterns of Cs_2_SnCl_6_ (JCPDS 75–0376) and Cs_2_TeCl_6_ (JCPDS 75–0377). c) SEM and EDS‐mapping images of Cs_2_Sn_0.5_Te_0.5_Cl_6_. d) XPS survey spectra of Cs_2_SnCl_6_, Cs_2_Sn_0.95_Te_0.05_Cl_6_, Cs_2_Sn_0.5_Te_0.5_Cl_6_, and Cs_2_TeCl_6_ MCs. e) High‐resolution XPS spectra of Cs, Sn, Te, and Cl elements in Cs_2_Sn_0.95_Te_0.05_Cl_6_.

Because Te element has similar electronic configurations with that of Sn, Te^4+^ cations with a wide range of concentration can be incorporated into Cs_2_SnCl_6_ matrix without causing significant change in structural phase. Moisture‐stable Cs_2_Sn_1−_ *_x_* Te*_x_* Cl_6_ (0 ≤ *x* ≤ 1) microcrystals (MCs) were first synthesized by a hydrochloric acid‐assisted precipitation method (HAAPM) as described previously in the literature.^[^ ^11a^ ^]^ Crystal structures of the three representative samples of *x* = 0, 0.05, and 0.5 are shown in **Figure** **1**a, they adopt a vacancy‐ordered structure with isolated [SnCl_6_]^2−^ and [TeCl_6_]^2−^ octahedra, respectively. Powder X‐ray diffraction (PXRD) patterns (Figure 1b) of the as‐synthesized crystals confirm the successful synthesis of the reference Cs_2_SnCl_6_ and Cs_2_TeCl_6_ materials. The actual atomic concentration of Te in the crystals was determined by inductively coupled plasma‐optical emission spectrometry, as shown in Table S1 in the Supporting Information. It was found that the elemental ratio of Sn to Te is very close to that of the nominal value. Elemental maps of a typical Cs_2_Sn_0.5_Te_0.5_Cl_6_ sample acquired by scanning electron microscopy coupled with energy dispersive spectroscopy (SEM‐EDS) show a homogenous distribution of the comprising elements (Figure 1c). X‐ray photoelectron spectroscopy (XPS) was used to investigate the oxidation state of each element. As shown in Figure 1d and Figure S1 in the Supporting Information, the Te 3d signal can be clearly seen in all XPS survey spectra of Cs_2_Sn_1−_ *_x_* Te*_x_* Cl_6_ (0 < *x* ≤ 1), confirming the successful incorporation of the Te element into Cs_2_SnCl_6_. High‐resolution XPS spectra depicted in Figure 1e show peaks located at around 737.9, 724.0, 586.76, 576.3, 495.9, 487.5, 200.3, and 198.6 eV, which are assignable to Cs^+^ 3d_3/2_, Cs^+^ 3d_5/2_, Te^4+^ 3d_3/2_, Te^4+^ 3d_5/2_, Sn^4+^ 3d_3/2_, Sn^4+^ 3d_5/2_, Cl^−^ 2p_1/2_, and Cl^−^ 2p_3/2_, respectively, evidencing the +1 state for Cs, +4 states for both Te and Sn, and −1 state for Cl in the crystal.^[^ ^13^ ^]^ A closer look at the XPS spectra shows that the binding energy of Cs 3d remains unchanged with increasing Te content (Figure S2, Supporting Information), whereas that of Cl 2p and Sn 3d shifts to higher binding energy and that of Te 3d shifts to lower binding energy. These results suggest a possible charge transfer from Cl/Sn to Te.

**Figure 2:**
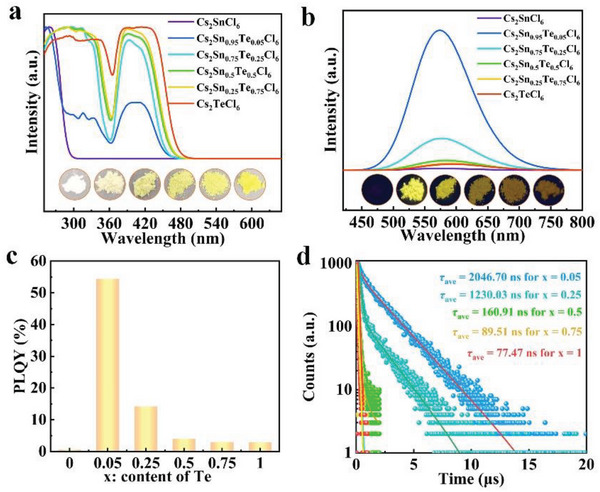
a) UV‐vis absorption spectra, photographs, and b) PL spectra of Cs_2_Sn_1−_ *_x_* Te*_x_* Cl_6_ MCs with different Te^4+^ concentrations. Inset shows photographs of the samples taken under 365 nm UV lamp. c) PLQY of the samples versus Te^4+^ concentration. d) TRPL spectra of Cs_2_Sn_1−_ *_x_* Te*_x_* Cl_6_ with different Te concentrations, monitored at 580 nm.

Influence of Te^4+^ incorporation on photophysical properties of Cs_2_SnCl_6_ is investigated by UV‐vis absorption and PL spectra. The optical absorption spectra of Cs_2_Sn_1−_ *_x_* Te*_x_* Cl_6_ samples are shown in **Figure** **2**a. The absorption edge of Cs_2_SnCl_6_ host is located around at 300 nm, which indicates a bandgap (*E*_g_) of around 4.39 eV, consistent with the literature value.^[^ ^10a^ ^]^ With the incorporation of Te^4+^, two distinguished absorption peaks appear between 300 and 360 nm and between 360 and 480 nm, which are gradually red‐shifted with increasing the *x* value. Previous studies have indicated that the additional absorption peak at longer wavelength (480 nm) is attributed to the bound excitons below the band gap, therefore, the absorption edges at around 360 nm should be used to determine their *E*_g_.^[^ ^10^, ^11^ ^]^ Density functional theory (DFT) calculations (Figure S3, Supporting Information) confirm that Cs_2_Sn_1−_ *_x_* Te*_x_* Cl_6_ (0.25 ≤ *x* ≤ 1) samples exhibit indirect band gap transition because of the filled pseudo‐closed Te 5s^2^ orbitals, while the Cs_2_Sn_1−_ *_x_* Te*_x_* Cl_6_ (0 ≤ *x* ≤ 0.05) samples exhibit direct band gap transition.^[^ ^10a^ ^]^ By Tauc plots (Figure S4, Supporting Information), *E*_g_ values of Cs_2_Sn_1−_ *_x_* Te*_x_* Cl_6_ samples can be thus determined, which decreased gradually with increasing the concentration of Te^4+^, in good line with their systematic color change (see inset of Figure 2a). PL spectra (excited at 380 nm) of the Cs_2_Sn_1−_ *_x_* Te*_x_* Cl_6_ crystals with varying *x* values are displayed in Figure 2b, which show a broad profile with maximum intensity at 575–580 nm. A slight red shift in the emission peak can be observed with increasing Te^4+^ concentration (Figure S5, Supporting Information), which coincides well with the change in *E*_g_.^[^ ^14^ ^]^ Of particular importance in PL observation is that a strongest PL emission was achieved when *x* = 0.05, whereas a severe PLQY deterioration from 55% to 3% was observed when further increasing *x* value from 0.05 to 1 (Figure 2c and Figure S6 in the Supporting Information, note that the pristine Cs_2_SnCl_6_ has no PL signal). Photographs (inset of Figure 2b) of Cs_2_Sn_1−_ *_x_* Te*_x_* Cl_6_ under 365 nm UV lamp exhibit the same trend as their PL spectra. Figure 2d shows the time‐resolved PL (TRPL) spectra of Cs_2_Sn_1−_ *_x_* Te*_x_* Cl_6_ (0 < *x* ≤1), monitored at 580 nm. Samples of *x* = 0.05, 0.25, and 0.5 can be well fitted by a bi‐exponential function, while crystals with *x* = 0.75 and 1 can be fitted by a single‐exponential function (Table S2, Supporting Information). Notably, PL lifetimes of Cs_2_Sn_1−_ *_x_* Te*_x_* Cl_6_ MCs shortened from 2046.7 to 77.5 ns as *x* value increases from 0.05 to 1, which complies well with the dramatical decrease in PLQY.

**Figure 3:**
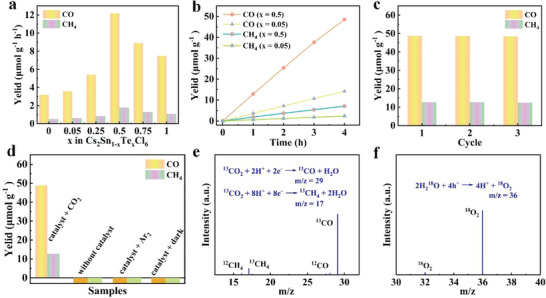
Photocatalytic CO_2_ reduction of Cs_2_Sn_1−_ *_x_* Te*_x_* Cl_6_ under simulated solar light. a) Comparison of production of CO and CH_4_ of Cs_2_Sn_1−_ *_x_* Te*_x_* Cl_6_. b) Time–yield plots of CO and CH_4_ over Cs_2_Sn_0.95_Te_0.05_Cl_6_ and Cs_2_Sn_0.5_Te_0.5_Cl_6_. c) Yields of CO and CH_4_ generated from CO_2_ reduction for three consecutive cycles (4 h per cycle) using Cs_2_Sn_0.5_Te_0.5_Cl_6_ as photocatalyst. d) Photocatalytic CO_2_ reduction performance of Cs_2_Sn_0.5_Te_0.5_Cl_6_ under different reaction conditions. e) Mass spectra of the ^13^CO and ^13^CH_4_ products after ^13^CO_2_ photoreduction over Cs_2_Sn_0.5_Te_0.5_Cl_6_. f) Mass spectra of the ^18^O_2_ product after H_2_^18^O photooxidation over Cs_2_Sn_0.5_Te_0.5_Cl_6_.

Cs_2_Sn_1−_ *_x_* Te*_x_* Cl_6_ perovskites with different concentrations of Te^4+^ were then employed as photocatalyst for photocatalytic CO_2_ reduction, which represents one of the most important and challenging artificial photosynthesis reactions.^[^ ^15^ ^]^ Notably, unlike the trend seen in PL emission, Cs_2_Sn_1−_ *_x_* Te*_x_* Cl_6_ perovskites exhibit improved photocatalytic performance when *x* value increases from 0.05 to 0.5, at which point significant PL quenching has occurred. The photocatalytic performance of Cs_2_Sn_1−_ *_x_* Te*_x_* Cl_6_ MCs toward CO_2_ reduction in the gas–solid interface was evaluated under AM 1.5G simulated solar irradiation, and the production rates of CO and CH_4_ are summarized and compared in **Figure** **3**a. When increasing Te^4+^ concentration to 50%, the photocatalytic performance reaches its maximum for CO (12.2 µmol g^−1^ h^−1^) and CH_4_ (1.8 µmol g^−1^ h^−1^) after 4 h irradiation, which is about 3.3 and 2.9 times higher than that of Cs_2_Sn_0.95_Te_0.05_Cl_6_ (3.6 µmol g^−1^ h^−1^ for CO, 0.62 µmol g^−1^ h^−1^ for CH_4_), respectively. Figure 3b shows the time‐dependent conversion yields of CO_2_ to CO and CH_4_ using Cs_2_Sn_0.5_Te_0.5_Cl_6_ and Cs_2_Sn_0.95_Te_0.05_Cl_6_ catalysts. Obviously, the performance of Cs_2_Sn_0.5_Te_0.5_Cl_6_ was significantly superior to that of Cs_2_Sn_0.95_Te_0.05_Cl_6_. Such a phenomenon can be well explained by considering the competition between the surface photocatalytic process and the recombination of the photogenerated carriers, i.e., the radiative recombination of the photogenerated carriers (i.e., PL process) in Cs_2_Sn_0.95_Te_0.05_Cl_6_ is more pronounced than that of Cs_2_Sn_0.5_Te_0.5_Cl_6_, therefore, we can expect a higher photocatalytic performance in the latter. Concerning stability of the catalyst, Cs_2_Sn_0.5_Te_0.5_Cl_6_ maintains a nearly linear increase in yield of CO and CH_4_, indicating its stable photocatalytic performance.^[^ ^16^ ^]^ Stability of the Cs_2_Sn_0.5_Te_0.5_Cl_6_ samples was further assessed by long‐term cycle test. As presented in Figure 3c, no significant decrease in photocatalytic activity was observed after three cycles of reaction (4 h per cycle), suggesting its excellent operational stability. Moreover, after a continuous photocatalytic reaction, no phase change takes place in the catalyst, as evidenced by XRD patterns (Figure S7, Supporting Information), which further confirms the excellent structural stability of Cs_2_Sn_0.5_Te_0.5_Cl_6_ in such a gas–solid photoreaction system. A background test was further performed to identify the key factors for CO_2_ reduction. Negligible CO and CH_4_ were detected in the absence of catalyst, CO_2_, and light (Figure 3d), implying that the simultaneous presence of the photocatalyst and light irradiation is essential for the photocatalytic CO_2_ reduction. Moreover, in an isotope tracer experiment using ^13^CO_2_ instead of CO_2_ as feeding gas, ^13^CO (*m*/*z* = 29) and ^13^CH_4_ (*m*/*z* = 17) were detected as the photocatalytic product (Figure 3e and Figure S8, Supporting Information), confirming that CO and CH_4_ were derived from the reduction of CO_2_. In addition, the isotope tracer experiment of ^18^O_2_ was designed by using H_2_^18^O as an alternative source to H_2_^16^O, ^18^O_2_ (*m*/*z* = 36) was detected (Figure 3f and Figure S9, Supporting Information), which demonstrates O_2_ was derived from the oxidation of H_2_O. The above findings suggest that a functional switch of Cs_2_SnCl_6_ perovskite between light emission and CO_2_ photocatalysis can be realized by controlling the amount of Te^4+^ in the lattice structure. Also, it is safe to conclude that unlike the doping scenarios for improving PL properties of Cs_2_SnCl_6_, a large amount of Te^4+^ incorporation is required for boosting their activity toward efficient photocatalytic CO_2_ reduction.

**Figure 4:**
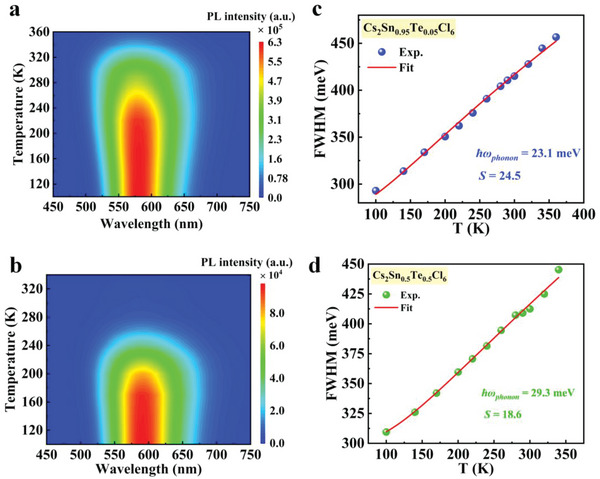
Temperature‐dependent emission properties. Pseudo‐color mapping of temperature‐dependent PL of a) Cs_2_Sn_0.95_Te_0.05_Cl_6_ and b) Cs_2_Sn_0.5_Te_0.5_Cl_6_. c,d) Fitting results of the FWHM as a function of temperature using the phonon broadening model.

To obtain a better understanding of the mechanism of the decreased PLQY and the improved photocatalytic performance when varying *x* value from 0.05 to 0.5, temperature‐dependent PL spectra were first measured. Previous studies have indicated that the strong PL emission of Cs_2_Sn_0.95_Te_0.05_Cl_6_ is mainly originated from [TeCl_6_]^2−^ octahedral enters, which emit light through Jahn–Teller‐like STEs due to the strong electron–phonon coupling in the halide matrix.^[^ ^10^, ^11^ ^]^ Since Huang‐Rhys factor (*S*) is a good measure of the strength of such coupling, we therefore monitored the change in *S* when *x* value increases from 0.05 to 0.5. The sum of *S* can be approximately derived based on the phonon broadening model using the following Equation (1)^[^ ^17^ ^]^

(1) $$\mathrm{FWHM}\;\left(T\right)=\;2.36\sqrt{S}\hbar \omega_{\mathrm{phonon}}\sqrt{\coth\frac{\hbar \omega_{\mathrm{phonon}}}{2k_{B}T}}$$

where FWHM is the PL full‐width at half‐maximum, *ħω* is the phonon frequency, and *k*_B_ is the Boltzmann constant. Figures S10 and S11 in the Supporting Information show the temperature‐dependent PL spectra of Cs_2_Sn_0.95_Te_0.05_Cl_6_ and Cs_2_Sn_0.5_Te_0.5_Cl_6_ in a temperature range 100–360 K, and the corresponding pseudo‐color maps are shown in **Figure** **4**a,b. FWHM data, derived from the temperature‐dependent PL spectra, are plotted in Figure 4c,d. FWHM increases with increasing temperature as a result of an enhanced optical phonon scattering. Nonlinear fitting of the curve using Equation (1) reveals that Cs_2_Sn_0.95_Te_0.05_Cl_6_ has *S* factor of 24.5, which confirms its strong electron–phonon coupling in the crystal.^[^ ^18^ ^]^ On the contrary, Cs_2_Sn_0.5_Te_0.5_Cl_6_ samples have *S* factor of 18.6, lower than that of Cs_2_Sn_0.95_Te_0.05_Cl_6_. This result indicates that the electron–phonon coupling becomes relatively weak at elevated levels of Te^4+^, which agrees well with the reduced PL intensity since formation of the emissive STEs is mainly due to the strong electron–phonon coupling. The weakened electron–phonon coupling also rationalizes the observation of the enhanced photocatalytic performance in Cs_2_Sn_0.5_Te_0.5_Cl_6_ because it renders photoexcited electrons easier to be dissociated, which thus facilitates charge transfer for surface photocatalytic CO_2_ reduction. Since phonons are quantized modes of vibration occurring in a rigid crystal lattice, we consider such change in electron–phonon coupling to be mainly caused by the local change in crystal structure after the incorporation of Te^4+^.^[^ ^7^ ^]^

**Figure 5:**
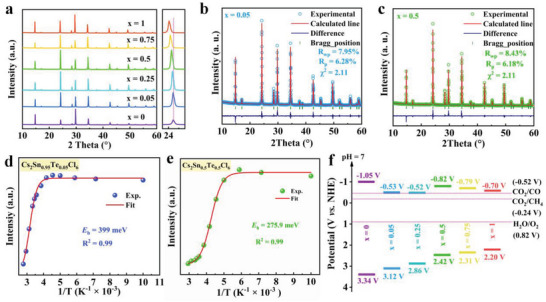
a) Powder XRD patterns of Cs_2_Sn_1−_ *_x_* Te*_x_* Cl_6_. Right panel is enlarged diffraction peak in the range of 24°–25°. XRD pattern and Rietveld refinement of b) Cs_2_Sn_0.95_Te_0.05_Cl_6_ and c) Cs_2_Sn_0.5_Te_0.5_Cl_6_. PL intensity versus 1/*T* and the linear fitting result of d) Cs_2_Sn_0.95_Te_0.05_Cl_6_ and e) Cs_2_Sn_0.5_Te_0.5_Cl_6_. f) Schematic band structure of Cs_2_Sn_1−_ *_x_* Te*_x_* Cl_6_.

To study the local structural evolution induced by Te^4+^ incorporation, XRD measurements were further performed on Cs_2_Sn_1−_ *_x_* Te*_x_* Cl_6_ samples with different Te^4+^ concentrations. **Figure** **5**a shows that the diffraction peaks of the Cs_2_Sn_1−_ *_x_* Te*_x_* Cl_6_ samples were shifted continuously toward lower angles with increasing the concentration of Te^4+^, which is indicative of a lattice expansion.^[^ ^19^ ^]^ This can be rationalized by the fact that Te^4+^ has a larger ionic radius (0.097 nm) than that of Sn^4+^ (0.069 nm). Rietveld refinement was further employed to resolve the detailed structural information after the incorporation of Te^4+^ (the as‐obtained goodness‐of‐fit parameter *χ* ^2^ is 2.11, and the reliability factor *R*_wp_ and *R*_p_ are around 8% and 6%, respectively). As shown in Figure 5b,c, the Rietveld analysis reveals that the lattice constant of the representative samples (Cs_2_Sn_0.95_Te_0.05_Cl_6_ and Cs_2_Sn_0.5_Te_0.5_Cl_6_) increases from 10.385 to 10.416 Å as increasing the concentration of Te^4+^ (Table S3, Supporting Information), which is consistent with the observed XRD peak shifts toward lower angles. The above results confirm a local structural evolution in Cs_2_SnCl_6_, making it reasonable for the Te^4+^ ions to induce a change in electron–phonon coupling in Cs_2_SnCl_6_. Further, we performed Raman measurement to identify the possible atomistic location of Te^4+^ ions in Cs_2_SnCl_6_. Te^4+^ ion can be incorporated as an interstitial and/or a substitutional dopant, both will cause lattice expansion and thus lead to XRD peak shift. As shown in Figure S12 in the Supporting Information, for the pristine Cs_2_SnCl_6_, the Raman shifts at 232 and 308 cm^−1^ are assignable to the Sn─Cl bond stretching vibration in [SnCl_6_]^2−^ octahedra, corresponding to asymmetric and symmetric modes, respectively. After the incorporation of Te^4+^, no significant change in both peak position and linewidth can be observed, which is indicative of the fact that the Te^4+^ ion preferentially occupies the substitutional site, rather than an interstitial site, because the latter case will usually induce an obvious lattice distortion and hence affect the Raman spectra of crystal lattice vibrational modes.^[^ ^20^ ^]^

In addition to the change in FWHM, PL spectra of the Cs_2_Sn_0.95_Te_0.05_Cl_6_ and Cs_2_Sn_0.5_Te_0.5_Cl_6_ crystals also undergo a continuous change in intensity. It is seen from Figure 5d,e that the integral PL intensity of Cs_2_Sn_0.95_Te_0.05_Cl_6_ gradually decreased from 140 to 300 K, accompanied by a shortened PL lifetime (Figure S13, Supporting Information). This can be due to the increased thermally populated vibrational states at high temperatures. The temperature dependence of the integrated PL intensity can be fitted by the Arrhenius formula (Equation (2)), from which one can obtain crystal's exciton binding energy, *E*_b_^[^ ^21^ ^]^

(2) $$I\;\left(T\right)=I_{0}/\left(1+Ae^{\frac{E_{b}}{k_{B}T}}\right)$$

where *I*(*T*) is the integrated PL intensity at *T* (K) and *A* is a constant. It is found that the Cs_2_Sn_0.95_Te_0.05_Cl_6_ single crystal possesses *E*_b_ of 399 meV, while that of Cs_2_Sn_0.5_Te_0.5_Cl_6_ has *E*_b_ value of 275.9 meV. The relatively weak exciton binding energy indicates that the excitons in Cs_2_Sn_0.5_Te_0.5_Cl_6_ become less localized compared to Cs_2_Sn_0.95_Te_0.05_Cl_6_. This can be understood by the fact that, under a low concentration of Te^4+^, the formed [TeCl_6_]^2‒^ octahedra in CsSnCl_3_ lattice are well isolated from each other by [SnCl_6_]^2‒^ octahedra, leading to enhanced exciton localization; increasing the amount of Te^4+^ in host makes the isolated [TeCl_6_]^2‒^ transfer to cluster, which weakens electronic confinement. The reduced exciton binding energy results in passive influence to charge interaction and hence makes the carriers more prone to nonradiative transition, which partially explains the decreased PLQY in Cs_2_Sn_0.5_Te_0.5_Cl_6_.^[^ ^22^ ^]^ On the other side, the reduced exciton binding energy also makes the excitons easier to be transferred to CO_2_, which helps to promote the surface photocatalytic reduction. To make a more detailed comparison of the exciton binding energy between Cs_2_Sn_1−_ *_x_* Te*_x_* Cl_6_ and other halide perovskites, we have included several representative samples in Table S4 in the Supporting Information.

Accompanied by the local change in crystal structure, we also found that Cs_2_Sn_1−_ *_x_* Te*_x_* Cl_6_ MCs have undergone significant change in micromorphology. SEM image shows that the grain size of Cs_2_Sn_1−_ *_x_* Te*_x_* Cl_6_ MCs was gradually decreased with increasing Te^4+^ concentration when *x* = 0–0.5 (Figures S14 and S15, Supporting Information). Such decrease in particle size leads to an increase in specific surface area, which is beneficial for enhancing their catalytic performance due to the increase in the availability of catalytic sites. Interestingly, our experimentally observed change in particle size is consistent with the theoretically calculated results in Gibbs free energy (Δ*G*), which suggest that Cs_2_Sn_1−_ *_x_* Te*_x_* Cl_6_ MCs have a minimum Δ*G* value when *x* = 0.5.^[^ ^11a^ ^]^ Our finding thus experimentally confirms that Cs_2_Sn_0.5_Te_0.5_Cl_6_ is the thermodynamically most stable phase among all compounds. Electronic band alignment of the studied catalysts is further examined as it is also a significant factor that influences their photocatalytic activity. Valence band maximum (VBM) of the Cs_2_Sn_1−_ *_x_* Te*_x_* Cl_6_ samples is determined by XPS valence band spectra. As displayed in Figure S16 in the Supporting Information, the valence band positions of Cs_2_Sn_1−_ *_x_* Te*_x_* Cl_6_ are gradually decreased with increasing the concentration of Te^4+^. This has been rationalized by previous DFT calculations which revealed that Te‐s orbits contribute to VBM of the crystals.^[^ ^10^, ^11^ ^]^ The corresponding conduction band minimum (CBM) can be therefore calculated based on the *E*_g_ value following the equation: *E*_CBM_ = *E*_VBM_ ‒ *E*_g_. Figure 5f shows the energy level alignments of CBM and VBM with various Te^4+^ concentrations. All of the samples possess the kinetically favorable band edge positions to realize the photoreduction of CO_2_. However, it is important to note that the energy difference between CBM and redox potential of CO_2_ reduction reaction in Cs_2_Sn_0.5_Te_0.5_Cl_6_ is largest among all catalysts, which means that it has the largest driving force for the electrons to transfer from catalyst to CO_2_ and thus being capable of yielding the highest catalytic performance.

**Figure 6:**
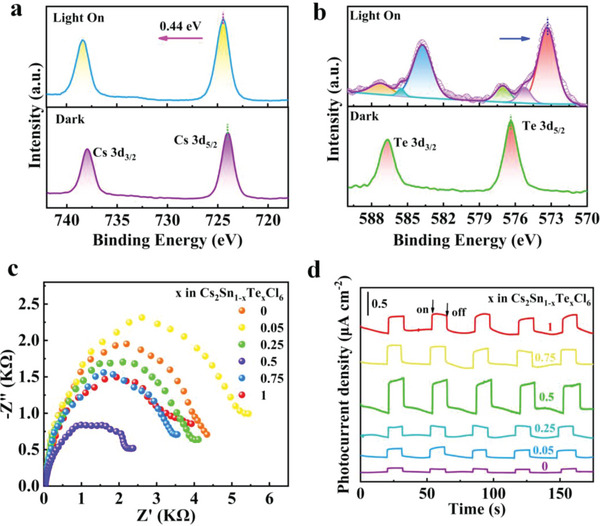
High‐resolution XPS spectra of the a) Cs 3d and b) Te 3d of Cs_2_Sn_0.5_Te_0.5_Cl_6_ tested in dark condition and under light irradiation. c) EIS and d) photocurrent response of Cs_2_Sn_1−_ *_x_* Te*_x_* Cl_6_ under simulated solar light (note that the vertical bar at the upper‐left corner denotes photocurrent intensity).

To determine the active sites on Cs_2_Sn_0.5_Te_0.5_Cl_6_, in situ XPS spectra measured under light irradiation were compared to that measured in dark. Upon in situ light irradiation, obvious positive shifts by 0.44, 0.65, and 0.57 eV were observed for the XPS spectra of Cs 3d, Sn 3d, and Cl 2p, respectively (**Figure** **6**a and Figure S17a,b, Supporting Information). In contrast, the peaks of Te 3d negatively shift from 576.3 to 573.3 eV (Figure 6b). Such variation in XPS binding energy after light irradiation indicates photogenerated electrons transferred from Cs, Sn, and Cl to Te, which proves that the Te site acts as the catalytically active site.^[^ ^23^ ^]^ Since photocatalytic performance of a catalyst is closely correlated with the charge transfer process, the kinetics of the photogenerated carriers in various Cs_2_Sn_1−_ *_x_* Te*_x_* Cl_6_ samples were studied by the electrical impedance technique to gain more insight into the improved catalytic performance in Cs_2_Sn_0.5_Te_0.5_Cl_6_. The electrochemical impedance spectra (EIS) were measured under 300 W Xe lamp irradiation. As shown in Figure 6c, samples of Cs_2_SnCl_6_ and Cs_2_Sn_0.95_Te_0.05_Cl_6_ display a relatively larger semicircle in the Nyquist plot, signifying that they have a larger resistance for charge transfer. On the contrary, the Cs_2_Sn_0.5_Te_0.5_Cl_6_ samples show the smallest semicircle, which benefits fast charge transport of the photogenerated carriers.^[^ ^24^ ^]^ Photocurrent response of the Cs_2_Sn_1−_ *_x_* Te*_x_* Cl_6_ samples was further tested under simulated solar light (Figure 6d). The photocurrent density of Cs_2_Sn_0.5_Te_0.5_Cl_6_ was obviously higher than that of the rest catalysts, which further confirms their better charge transport ability and thus higher capability for efficient CO_2_ photocatalytic reduction.

**Figure 7:**
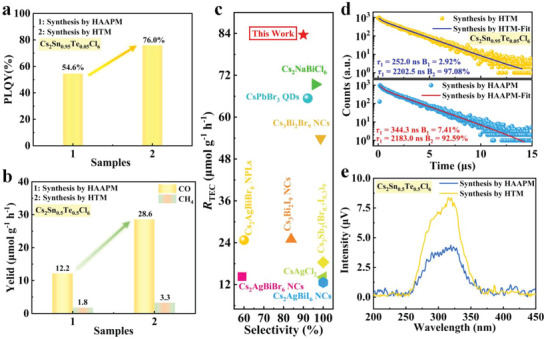
a) Comparison on PLQY of Cs_2_Sn_0.95_Te_0.05_Cl_6_ and b) photocatalytic performance of Cs_2_Sn_0.5_Te_0.5_Cl_6_ synthesized by HAAPM and hydrothermal method (HTM). c) Summary of the reported single‐component halide perovskites for photocatalytic CO_2_ reduction, *R*_TEC_ = *R*_CO_ × 2 + *R*_CH4_ × 8. d) TRPL results of Cs_2_Sn_0.95_Te_0.05_Cl_6_ and e) steady‐state surface photovoltage spectra of Cs_2_Sn_0.5_Te_0.5_Cl_6_ synthesized by HAAPM and HTM, respectively.

The PLQY of Cs_2_Sn_0.95_Te_0.05_Cl_6_ and the performance for photocatalytic CO_2_ reduction of Cs_2_Sn_0.5_Te_0.5_Cl_6_ were further improved by optimizing the synthesis method. As shown in **Figure** **7**a, the PLQY of Cs_2_Sn_0.95_Te_0.05_Cl_6_ synthesized by HTM was increased to 76%, exceeding that of Cs_2_Sn_0.95_Te_0.05_Cl_6_ synthesized by HAAPM (Figure S18 and Table S5, Supporting Information). Similarly, compared to the photocatalytic performance (12.2 µmol g^−1^ h^−1^ for CO and 1.8 µmol g^−1^ h^−1^ for CH_4_) of Cs_2_Sn_0.5_Te_0.5_Cl_6_ synthesized by HAAPM, the activity of photocatalytic CO_2_ reduction was enhanced to 28.6 µmol g^−1^ h^−1^ for CO and 3.3 µmol g^−1^ h^−1^ for CH_4_ (Figure 7b). Moreover, the selectivity of CO reaches as high as ≈90%. Further, we made a comparison of some recently reported single‐component halide perovskites for photocatalytic CO_2_ reduction (Figure 7c). The total electron consumption (*R*_TEC_) of our Cs_2_Sn_0.5_Te_0.5_Cl_6_ reaches 83.6 µmol g^−1^ h^−1^, which is superior to the performance of most reported halide perovskites.^[^ ^25^ ^]^ The improved catalytic activity can be attributed to the decreased bulk defect density in HTM‐prepared sample. Normally, the solubility of the precursor salt in hydrochloric acid increases under high temperature and pressure conditions. As a result, the generation and growth of nucleus of halide perovskite would be slow during the cooling process, which favors the formation of semiconductor materials with lower defect density. In contrast, for those Cs_2_Sn_0.5_Te_0.5_Cl_6_ synthesized by HAAPM, because the reaction runs under ambient conditions, the nucleation and growth of perovskite is rapid, which can easily generate defects. To confirm this, TRPL and surface photovoltage spectra measurements were further conducted. As shown in Figure 7d, the PL decay curves of Cs_2_Sn_0.95_Te_0.05_Cl_6_ synthesized by HAAPM and HTM can be well fitted by a bi‐exponential function. Cs_2_Sn_0.95_Te_0.05_Cl_6_ synthesized by HTM has a significantly smaller percentage of shorter lifetimes than that synthesized by HAAPM, which means that the density of defects in the former is lower than that in the latter. In addition, the surface photovoltage of Cs_2_Sn_0.5_Te_0.5_Cl_6_ synthesized by HTM is higher than that synthesized by HAAPM (Figure 7e), confirming a lower density of defects in HTM‐prepared samples. The reduction in defect density retards the charge recombination so that charge carriers would have more chance to participate in the following catalytic reaction.

**Figure 8:**
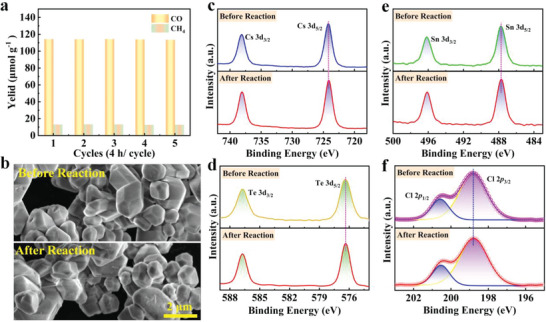
a) Yields of CO and CH_4_ generated from CO_2_ reduction for five consecutive cycles (4 h per cycle) using HTM‐synthesized Cs_2_Sn_0.5_Te_0.5_Cl_6_. b) SEM images, c) high‐resolution XPS spectra of Cs 3d, d) Sn 3d, e) Te 3d, and Cl 2p of the catalysts before and after the photocatalytic reaction.

Stability of the Cs_2_Sn_0.5_Te_0.5_Cl_6_ samples synthesized by HTM was first assessed by long‐term cycle test. No significant decrease in photocatalytic activity was observed after five cycles of reaction (**Figure** **8**a), indicative of its excellent operational stability. Moreover, after a continuous photocatalytic reaction, the catalyst maintains good structural morphology, as evidenced by SEM (Figure 8b), further confirming the excellent structural stability of Cs_2_Sn_0.5_Te_0.5_Cl_6_. The high‐resolution XPS spectra (Figure 8c–f) of Cs_2_Sn_0.5_Te_0.5_Cl_6_ catalysts show that the peak positions of Cs 3d, Sn 3d, Te 3d, and Cl 2p all remain intact after the photocatalytic reaction, indicating that the valence states of these comprising elements are not changed, which also confirms the excellent chemical stability of Cs_2_Sn_0.5_Te_0.5_Cl_6_ in such a gas–solid photoreaction system.

## 3 Conclusion

In summary, Te^4+^‐incorporated Cs_2_SnCl_6_ was used for the first time as catalyst for CO_2_ reduction. By controlling the amount of Te^4+^ ion in Cs_2_SnCl_6_, we can readily switch the functions of Cs_2_SnCl_6_ from light emission to CO_2_ photocatalysis. Under a low concentration of Te^4+^, the formed [TeCl_6_]^2‒^ octahedra tend to be isolated from each other, resulting in a strong charge localization, which makes them suitable for light emission; a further increase in concentration of Te^4+^ weakens charge localization, which reduces exciton binding energy, as well as the electron–phonon coupling, facilitating charge separation and thus the charge transfer to CO_2_ for efficient photocatalytic reduction. In addition, the increase in Te^4+^ concentration also leads to a significant change in both particle size and energy band position of Cs_2_SnCl_6_, which, combined with the improvement in charge delocalization, exert a favorable synergistic effect on the performance of CO_2_ photocatalysis. The results of this study provide access to new CO_2_ photocatalysts and also provide insights into the versatility of all‐inorganic, Pb‐free perovskites.

## Conflict of Interest

The authors declare no conflict of interest.

## Supporting information

Supporting Information

## Data Availability

The data that support the findings of this study are available in the Supporting Information of this article.
