# Supplementary material for: Cs2SnCl6: To Emit or to Catalyze? Te4+ Ion Calls the Shots
Source: Adv Sci (Weinh). 2023 Aug 9;10(29):2302706. doi: 10.1002/advs.202302706 (PMC10582433; doi:10.1002/advs.202302706)
Supplement: Supplementary file 1 — Supporting Information [file ADVS-10-2302706-s001.pdf]

ADVANCED SCIENCE
Open Access

Supporting Information

for *Adv. Sci.*, DOI 10.1002/advs.202302706

Cs_2SnCl_6: To Emit or to Catalyze? Te^{4+} Ion Calls the Shots

Haiwen Wei, Jikai Sun, Xin Mao, Honglei Wang, Zhen Chen, Tianxin Bai, Pengfei Cheng, Ruiling Zhang, Bing Jin, Panwang Zhou, Feng Liu and Keli Han*

Supporting Information

Cs_2SnCl_6: To emit or to catalyze? Te^{4+} ion calls the shots

Haiwen Wei [a], *Jikai Sun* [a], *Xin Mao* [a], *Honglei Wang* [b], *Zhen Chen* [a], *Tianxin Bai* [a], *Pengfei Cheng* [c], *Ruiling Zhang* [a], *Bing Jin* [a], *Panwang Zhou* [a], *Feng Liu** [a], *Keli Han*+ [a c]

a - Institute of Molecular Sciences and Engineering, Institute of Frontier and Interdisciplinary Science, Shandong University, Qingdao 266237, P. R. China

b - School of Chemical Engineering, Dalian University of Technology, Dalian 116024, P. R. China

c - State Key Laboratory of Molecular Reaction Dynamics, Dalian Institute of Chemical Physics, Chinese Academy of Science, Dalian 116023, P. R. China

1. Experimental Section

1.1 Materials

All reagents were used as received without further purification: Cesium chloride (CsCl, Aladdin, 99.9%), stannic chloride ($SnCl_4$, anhydrous, 99%, Alfa Aesar), telluric chloride ($TeCl_4$, 99.9%, Alfa Aesar), hydrochloric acid (HCl, analytical pure, Sinopharm Chemical Reagent Co., Ltd, China).

1.2 Synthesis of $Cs_2Sn_{1-x}Te_xCl_6$ ($0 \leq x \leq 1$) by hydrochloric acid-assisted precipitation method (HAAPM)

For $Cs_2Sn_{1-x}Te_xCl_6$ (x = 0, 0.05, 0.25, 0.5, 0.75, 1), x mmol of $TeCl_4$ and 1-x mmol of $SnCl_4$ were dissolved in 5 mL HCl for A solution, and 2 mmol of CsCl is dissolved in 2 mL HCl for B solution. Then the B solution is rapidly injected into A solution with vigorously stirring, and the $Cs_2Sn_{1-x}Te_xCl_6$ is precipitated at the bottom. Finally, the obtained powder (undoped sample: white color; doped sample: yellowish color) was filtered out and washed with ethanol before drying at 60 ℃ overnight.

1.3 Synthesis of $Cs_2Sn_{1-x}Te_xCl_6$ (x = 0.05 and 0.5) by hydrothermal method (HTM)

Preparation method for solution A, B is same as described in section 1.2. The two solutions were mixed together and then transferred to a polytetrafluoroethylene lined reactor. After reaction at 180 °C for 12 h, the reactor was allowed to be slowly cooled to room temperature. The obtained powder was filtered out and washed with ethanol before drying at 60 ℃ overnight.

1.4 Characterization

Powder X-ray diffraction (XRD) measurement was performed on a PANalytical Empyrean diffractometer equipped with Cu Kα X-ray (λ =1.54056 Å) tubes, and the acquisition was done for every 0.05° increment. Inductively coupled plasma optical emission spectrometer (ICP-OES) was performed on PerkinElmer ICP-OES 7300DV. The scanning electron microscopy (SEM) measurements were performed by using the Quanta 250 FEG. Steady-state absorption spectra were recorded using a UV-vis (SHIMADZU UV2600) spectrometer. Optical diffuse reflectance measurement was performed by equipping with an integrating sphere at room temperature and $BaSO_4$ as the 100% reflectance reference. The reflectance data were converted to absorption according to the Kubelka-Munk equation. The chemical states of the samples were determined by X-ray photoelectron spectroscopy (XPS) on ESRCALAB250Xi, Thermo Fisher Scientific. The binding energies are referenced to the C 1s peak at the binding energy of 284.8 eV. PLQY were measured by using an integrating sphere on an Absolute PL Quantum Yield Spectrometer (C9920-02G, Japan). Steady-state PL and time-resolved PL (TRPL) measurements were performed on a FLS1000 spectrofluorimeter (Edinburgh Instruments Ltd, UK) with 380 nm excitation wavelength. The TRPL spectra were recorded using time-correlated single photon counting (TCSPC) technology and fitted with di-exponential function (for x = 0.05, 0.25 and 0.5) and single-exponential function (for x = 0.75 and 1). The average PL lifetimes (τ_{ave}) were calculated as follows:

$$A(t) = A_1 e^{(-t/\tau 1)} + A_2 e^{(-t/\tau 2)}$$

$$\tau_{ave} = (A_1\tau_1^2 + A_2\tau_2^2)/(A_1\tau_1 + A_2\tau_2)$$

The electrochemical impedance spectroscopy (EIS) was carried out using a three-electrode cell (CHI 660D, Shanghai Chenhua) with a Pt foil counter electrode and a saturated Ag/AgCl reference electrode at the open circuit potential using a frequency ranged from 10^6 Hz to 10^{-1} Hz. The working electrode is prepared by dip-coating method. About 10 mg of the photocatalyst is dispersed in 1.5 mL of acetonitrile and 10 μL Nafion solution to form slurry. Then, 4 μL of the slurry was dip-coated on the glassy carbon electrode. Acetonitrile solution with 0.1 mol/L of tetrabutylammonium hexafluorophosphate ($TBAPF_6$) was used as the electrolyte.

1.5 Photocatalytic CO_2 reduction

A solid-gas mode was adopted to evaluate the photocatalytic CO_2 reduction activity of $Cs_2Sn_{1-x}Te_xCl_6$ (x = 0, 0.05, 0.25, 0.5, 0.75, 1). The measurements were performed within a 50 mL sealed Pyrex bottle filled with CO_2 and H_2O vapor. Specifically, the vacuum treated clean sample films (catalyst mass of about 10 mg) and 30 μL water were put into the bottle which

was degassed repeatedly to remove air and then charged with CO_2. Then the sealed Pyrex bottle was heated at 120 °C to accelerate the evaporation of water. A 300 W Xe lamp (PLS-SXE300+, Beijing Perfectlight Technology Co., Ltd) equipped with a standard AM 1.5G filter was utilized as the solar light source. The obtained gaseous product was qualitatively analyzed by gas chromatograph (Agilent 8890) equipped with a thermal conductivity detector (TCD) and a flame ionization detector (FID) in series. The isotope-labeling experiment of $^{13}CO_2$ and $^{18}O_2$ were analyzed by GC-MS (Thermo Fisher-TSQ9000).

2. Theoretical Methods

All theoretical calculations were performed using DFT with the projector augmented wave (PAW) method[1], as implemented in the Vienna Ab Initio Simulation Package[2] (VASP). The Perdew–Burke–Ernzerhof (PBE) functional based on the generalized gradient approximation (GGA) was chosen to account for the exchange-correlation energy[3]. The kinetic energy cut-off of the plane wave basis set was set to 500 eV. A The van der Waals interaction was considered using Grimme's DFT-D3 correction method[4]. Geometries were assumed to be converged when Hellmann-Feynman forces on each atom were less than 0.01 eV/Å. The 5 × 5 × 1 Monkhorst-Pack k-points grid was utilized for Brillouin zone sampling with an energy convergence threshold of 10^{-5} eV. Electronic structure analyses were performed by the python library[5] (pymatgen).

Supplementary Figures and Tables

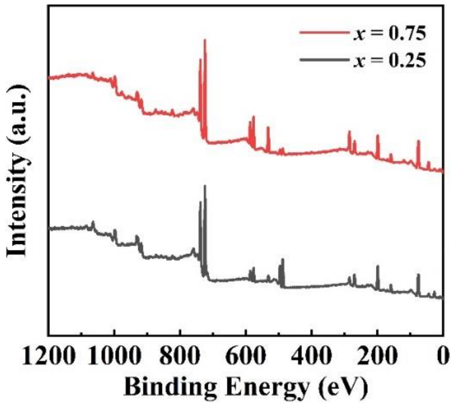

Figure S1. XPS survey spectra of $Cs_2Sn_{0.75}Te_{0.25}Cl_6$ and $Cs_2Sn_{0.25}Te_{0.75}Cl_6$ MCs.

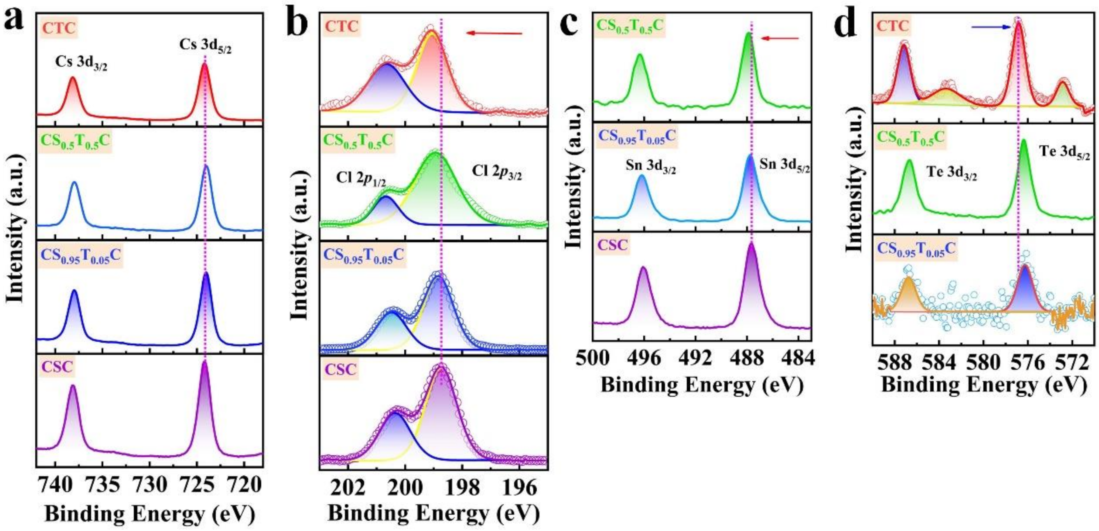

Figure S2. High-resolution XPS spectra of (a) Cs, (b) Cl, (c) Sn, and (d) Te for $Cs_2Sn_{1-x}Te_xCl_6$ (x = 0, 0.05, 0.5, 1).

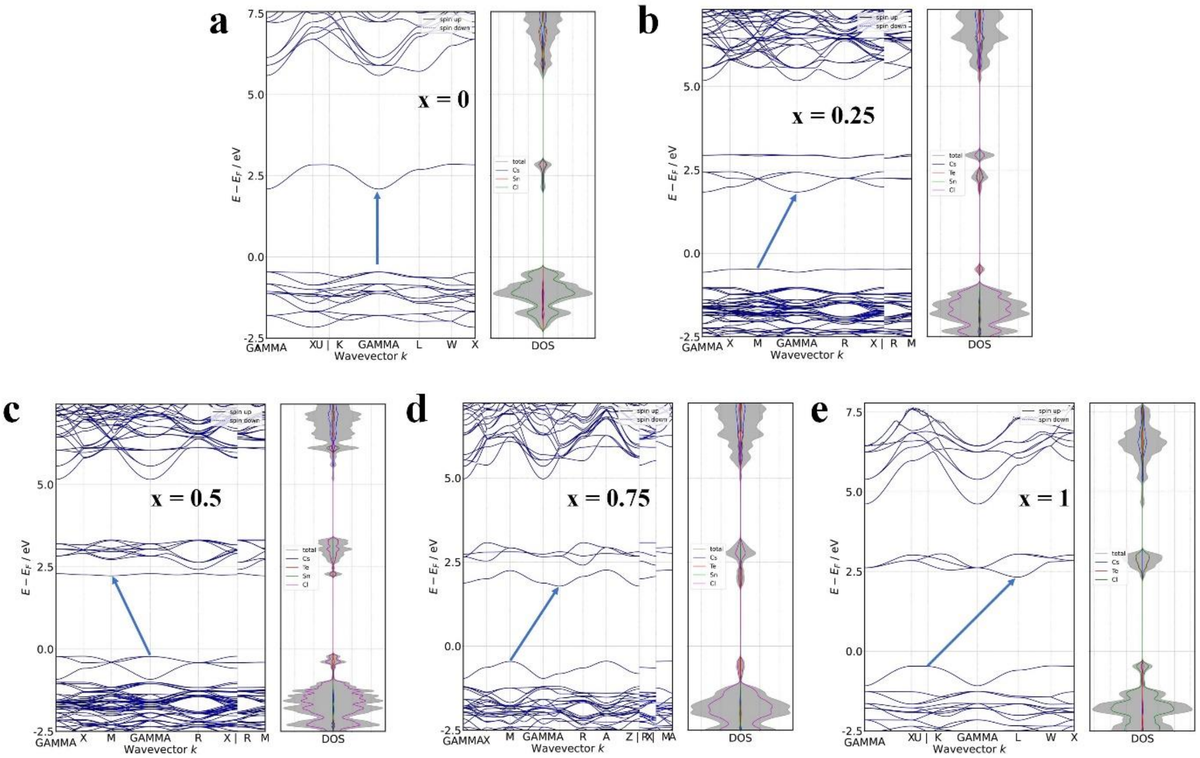

Figure S3. Calculated band structures and the DOS of $Cs_2Sn_{1-x}Te_xCl_6$ (x = 0, 0.25, 0.5, 0.75, 1)

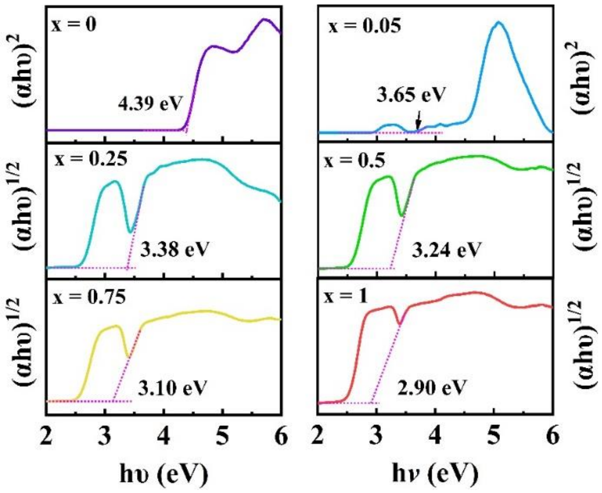

Figure S4. Tauc plots of the transformed Kubelka-Munk function versus photon energy of $Cs_2Sn_{1-x}Te_xCl_6$.

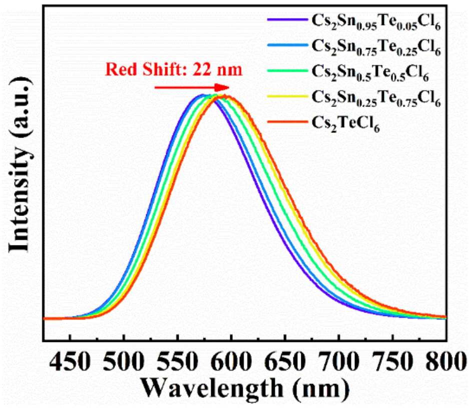

Figure S5. PL spectra of $Cs_2Sn_{1-x}Te_xCl_6$ ($0 < x \leq 1$), excited at 380 nm.

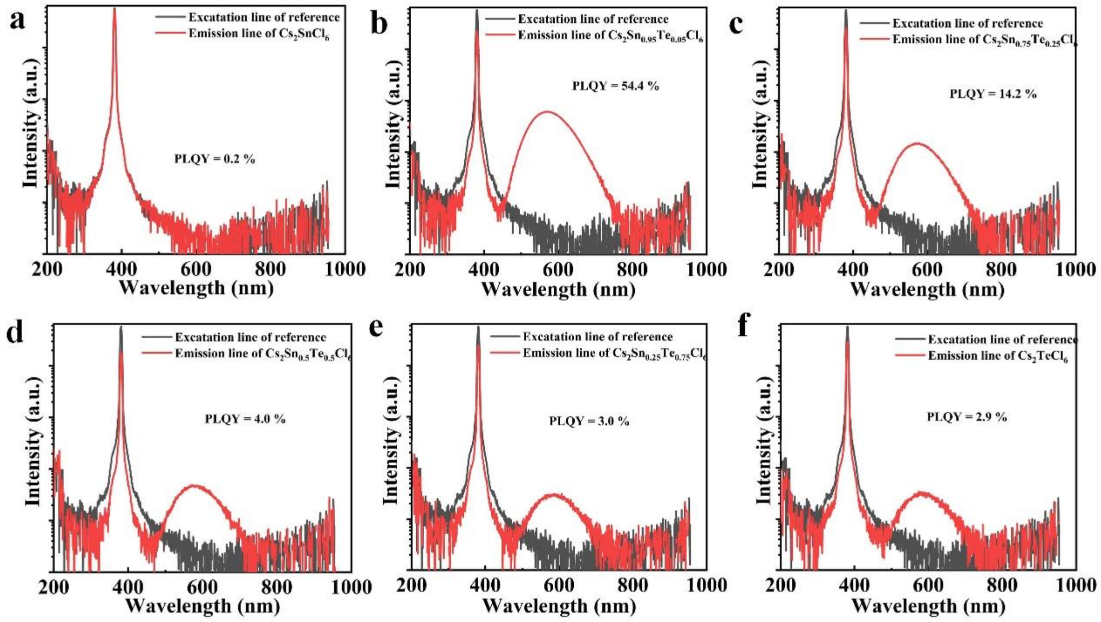

Figure S6. Characterization of the PLQYs of $Cs_2Sn_{1-x}Te_xCl_6$, including their excitation spectra of reference and emission spectra.

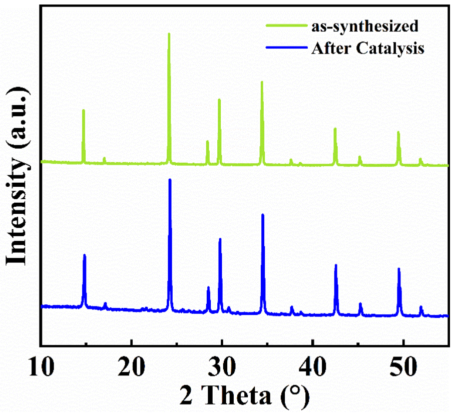

Figure S7. XRD patterns of $Cs_2Sn_{0.5}Te_{0.5}Cl_6$ before and after catalysis.

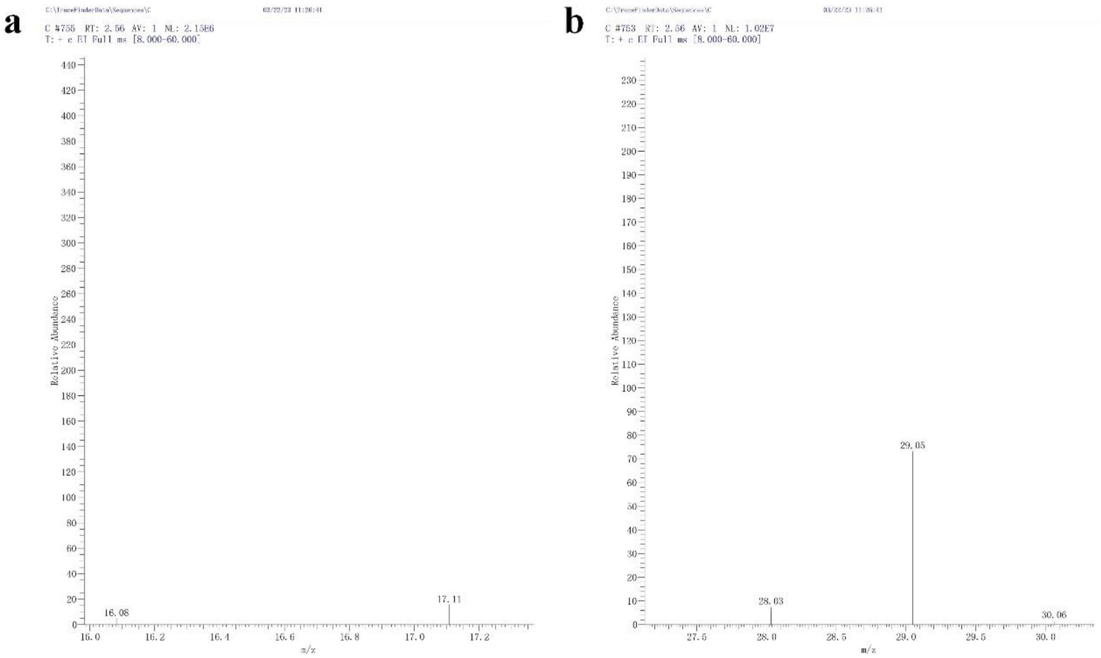

Figure S8. Mass spectra of reaction products of photocatalytic CO_2 reduction using $Cs_2Sn_{0.5}Te_{0.5}Cl_6$ as catalyst. The peaks at m/z = 16, m/z = 17, m/z = 28 and m/z = 29 can be assigned to $^{12}CH_4$, $^{13}CH_4$, ^{12}CO, and ^{13}CO, respectively.

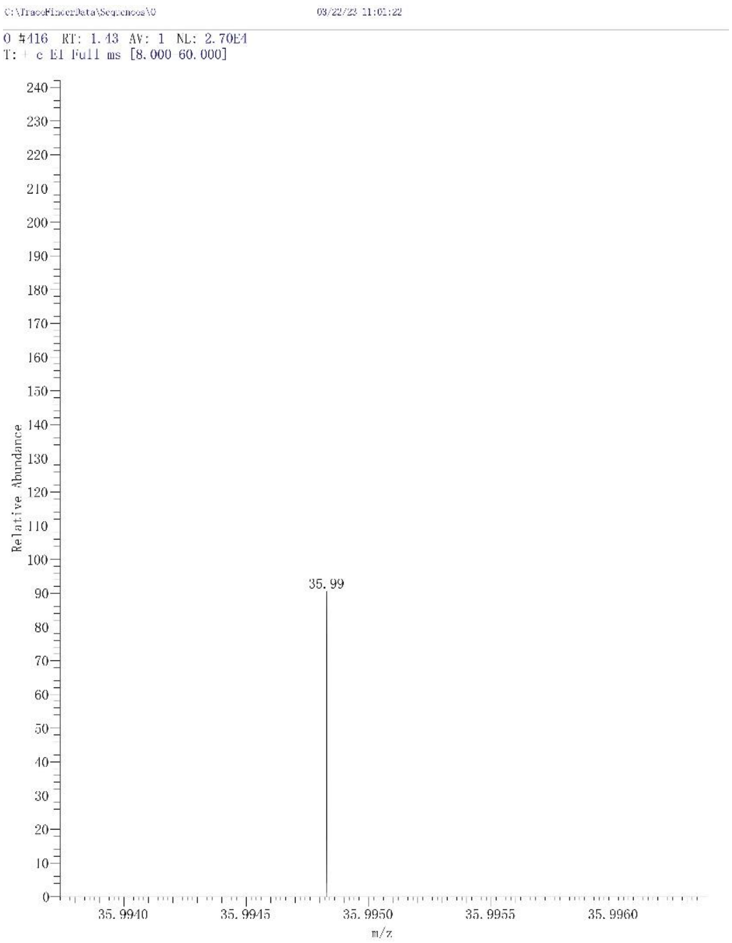

Figure S9. Mass spectra of reaction products of photocatalytic CO_2 reduction using $Cs_2Sn_{0.5}Te_{0.5}Cl_6$ as catalyst. The peaks at *m*/*z* = 36 can be assigned to $^{18}O_2$.

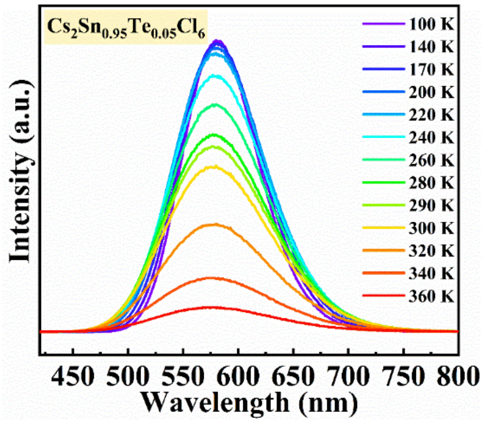

Figure S10. Temperature-dependent emission properties of $Cs_2Sn_{0.95}Te_{0.05}Cl_6$.

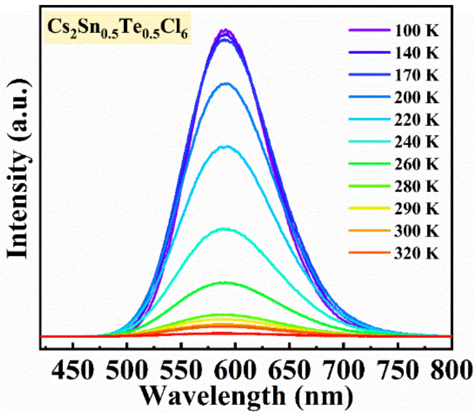

Figure S11. Temperature-dependent emission properties of $Cs_2Sn_{0.5}Te_{0.5}Cl_6$.

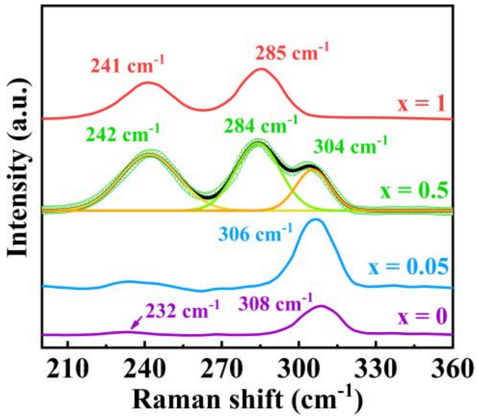

Figure S12. Raman spectra of Cs_2SnCl_6, $Cs_2Sn_{0.95}Te_{0.05}Cl_6$, $Cs_2Sn_{0.5}Te_{0.5}Cl_6$, and Cs_2TeCl_6 MCs.

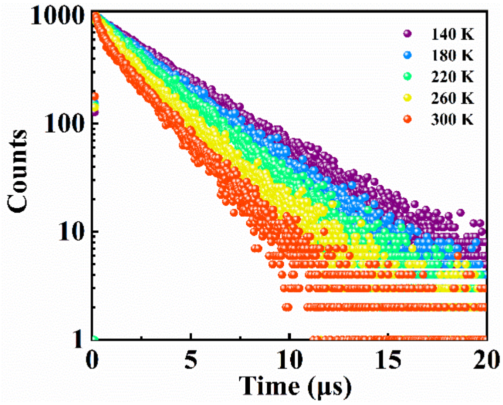

Figure S13. TRPL spectra of $Cs_2Sn_{0.95}Te_{0.05}Cl_6$, measured at different temperatures.

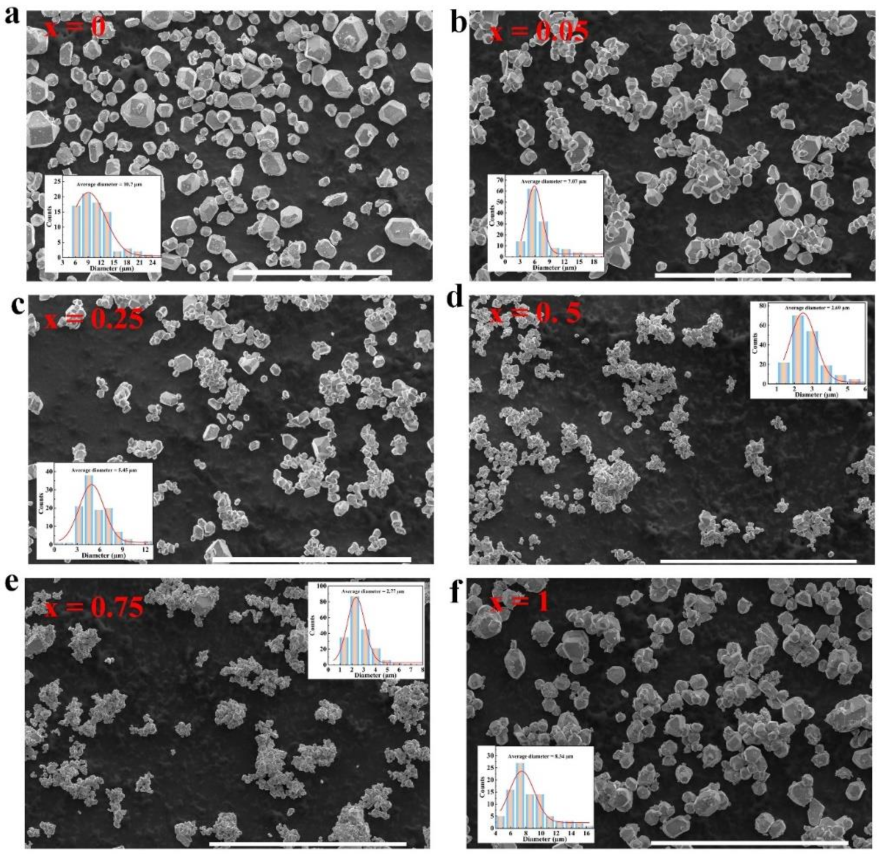

Figure S14. SEM images and particle size distribution of $Cs_2Sn_{1-x}Te_xCl_6$ ($0 \leq x \leq 1$).

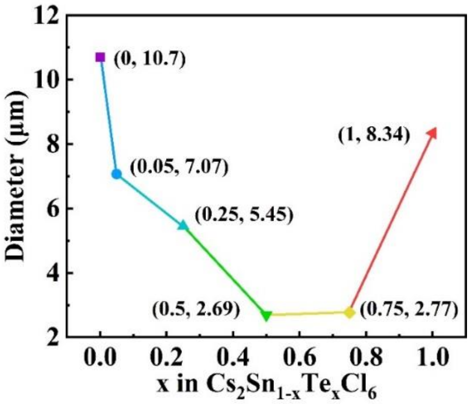

Figure S15. The trend of the average particle sizes of $Cs_2Sn_{1-x}Te_xCl_6$ with increasing Te^{4+} ion concentration.

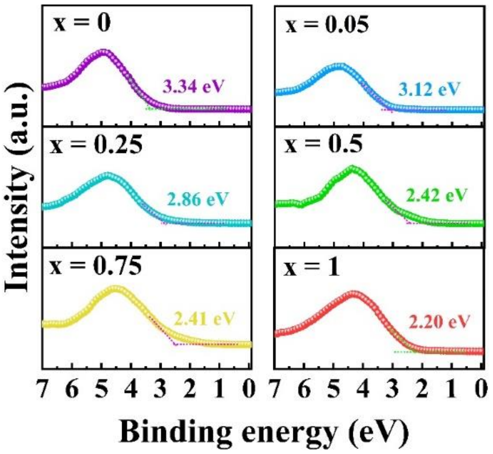

Figure S16. XPS valence band spectra of $Cs_2Sn_{1-x}Te_xCl_6$.

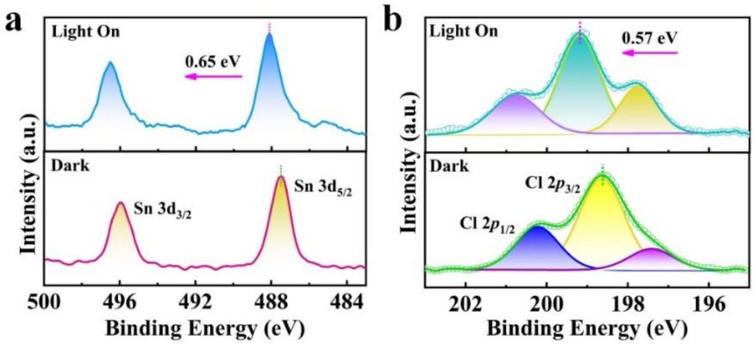

Figure S17. Comparison of high-resolution XPS spectra of (a) Sn 3*d* and (b) Cl 2*p* of $Cs_2Sn_{0.5}Te_{0.5}Cl_6$, tested in dark conditions and under in-situ light irradiation, respectively.

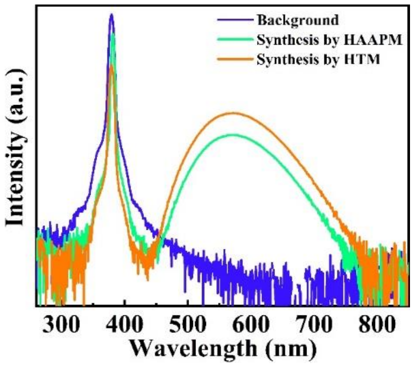

Figure S18. Characterization of the PLQYs of $Cs_2Sn_{0.95}Te_{0.05}Cl_6$ synthesized by HAAPM and HTM.

Table S1. Nominal and actual element ratios of Sn:Te in $Cs_2Sn_{1-x}Te_xCl_6$.

Sample	Nominal element ratio (Sn : Te)	Actual element ratio (Sn : Te)
$Cs_2Sn_{0.95}Te_{0.05}Cl_6$	0.95:0.05	0.953:0.047
$Cs_2Sn_{0.75}Te_{0.25}Cl_6$	0.75:0.25	0.78:0.22
$Cs_2Sn_{0.5}Te_{0.5}Cl_6$	0.5:0.5	0.51:0.49
$Cs_2Sn_{0.25}Te_{0.75}Cl_6$	0.25:0.75	0.25:0.75

Table S2. Structural parameters of $Cs_2Sn_{0.95}Te_{0.05}Cl_6$ and $Cs_2Sn_{0.5}Te_{0.5}Cl_6$.

	x = 0.05	x = 0.5
Crystal System	Cubic	Cubic
Space Group	Fm-3m	Fm-3m
a, b, c	10.385, 10.385, 10.385	10.416, 10.416, 10.416
α, β, γ	90, 90, 90	90, 90, 90
Volume (Å^3)	1120.263	1130.233
Sn-Cl (Å)	2.55683	2.56445
Te-Cl (Å)	2.55683	2.56445

Table S3. Decay lifetimes of TRPL and relative amplitudes of photoexcited charge carriers in $Cs_2Sn_{1-x}Te_xCl_6$.

Materials	τ_1 (ns)	A_1 (%)	τ_2 (ns)	A_2 (%)	τ_{ave} (ns)
$Cs_2Sn_{0.95}Te_{0.05}Cl_6$	344	7.41	2183	92.59	2046.7
$Cs_2Sn_{0.75}Te_{0.25}Cl_6$	189.9	36.72	1833.7	63.28	1230
$Cs_2Sn_{0.5}Te_{0.5}Cl_6$	110.4	88.39	545.3	11.61	160.9
$Cs_2Sn_{0.25}Te_{0.75}Cl_6$	89.5	100	/	/	89.5
Cs_2TeCl_6	77.5	100	/	/	77.5

Table S4. Summary of the exciton binding energy values of some representative halide perovskites.

Materials	E_b	Reference
$MAPbCl_{1-x}I_x$	50 meV	*Nat. Commun*. 2014, 5, 3586
$MAPbI_3$ single crystal	16 meV	*Nat. Phys*. 2015, 11, 582
$MAPbI_3$ films	35 meV	*J. Phys. Chem. Lett.* 2014, 13, 2189.
$Cs_2AgBiBr_6$	70 meV	*Adv. Mater.* 2018, 30, 1804450
$MAPbBr_3$	380 meV	*J. Phys. Chem. Lett.* 2018, 9, 4066.
$Cs_2Ag_{0.05}Na_{0.95}BiCl_6$	140 meV	*Angew. Chem. Int. Ed.* 2022, 33, e202207454
$Cs_3Sb_2Br_9$	530 meV	*ACS Nano* 2017, 9, 9294

Table S5. The PLQY of $Cs_2Sn_{0.95}Te_{0.05}Cl_6$ synthesized by HTM were tested five times.

Materials	Samples 1	Samples 2	Samples 3	Samples 4	Samples 5
$Cs_2Sn_{0.95}Te_{0.05}Cl_6$	75.6%	75.9%	76%	75.8%	75.8%

References

[1] P. E. Blöchl, *Phys. Rev. B* **1994**, 50, 17953.

[2] G. Kresse, J. Furthmüller, *Phys. Rev. B* **1996**, 54, 11169.

[3] a) J. P. Perdew, K. Burke, M. Ernzerhof, *Phys. Rev. B* **1996**, 77, 3865; b) J. P. Perdew, Y. Wang, *Phys. Rev. B* **1992**, 45, 13244; c) J. P. Perdew, J. A. Chevary, S. H. Vosko, K. A. Jackson, M. R. Pederson, D. J. Singh, C. Fiolhais, *Phys. Rev. B* **1992**, 46, 6671.

[4] L. Goerigk, S. Grimme, *Phys. Chem. Chem. Phys.* **2011**, 13, 6670.

[5] S. P. Ong, W. D. Richards, A. Jain, G. Hautier, M. Kocher, S. Cholia, D. Gunter, V. L. Chevrier, K. A. Persson, G. Ceder, *Comput. Mater. Sci.* **2013**, 68, 314.
